# Dutch and Indonesian teachers on teaching medical ethics: what are the learning goals?

**DOI:** 10.1080/10872981.2022.2079158

**Published:** 2022-05-23

**Authors:** Amalia Muhaimin, Maartje Hoogsteyns, Diyah Woro Dwi Lestari, Miko Ferine, Adi Utarini, Derk Ludolf Willems

**Affiliations:** a Department of Bioethics and Humanities, Faculty of Medicine, Universitas Jenderal Soedirman, Purwokerto, Indonesia; b Department of Ethics, Law, and Humanities, Amsterdam University Medical Center, University of Amsterdam, The Netherlands; c Amsterdam Public Health Research Institute, Amsterdam, The Netherlands; d Department of Health Policy and Management, Faculty of Medicine, Public Health and Nursing, Universitas Gadjah Mada, Yogyakarta, Indonesia

**Keywords:** Teachers, medical ethics, learning goals, Indonesia, Netherlands

## Abstract

Previous literature has discussed the different views, the diverse goals and scope of ethics education, and the need for a more homogenous curriculum in medical ethics. Since ethics is about values, and values are partly influenced by culture, we question to what extent teachers’ perceptions concerning learning goals of medical ethics curricula are similar or different in two different countries, and if differences in learning goals are acceptable or problematic. We conducted in-depth interviews with 36 medical ethics teachers, 20 from Indonesia and 16 from the Netherlands, and explored what they think are the important learning goals. We found three similar goals, with slightly different perceptions, between the two groups: (1) being professional, (2) dealing with ethical problems, and (3) being part of society. We also found four other goals that differed between the two countries: (4) *understanding one-self* and (5) *learning from others* from the Netherlands; (6) *being faithful/pious* and (7) *obeying rules/standards* from Indonesia. We suggest that despite similar goals shared globally, there might be differences in how teachers in different cultural contexts perceive the goals with their local values and translate them into the curricula. Differences in learning goals are common and natural, often reflected by historical and sociocultural contexts, and should not become a barrier for teachers in different regions to collaborate. Understanding these differences may be an important goal for teachers themselves to broaden their knowledge and perspectives.

## Background

Current developments in medical education have brought new educational frameworks for medicine in general and for ethics teaching in particular. Each country has its own framework that is often used as a starting point to develop their ethics curriculum. For instance, Dutch medical schools use the Medical Training Framework known as *Raamplan Artsopleiding* [1], while Indonesian medical schools use the Standard of Competencies for Indonesian Physicians (*SKDI*) [2]. Despite the thorough description of the scopes and expected competencies in clinical knowledge and skills, the scope and goals in ethics remain somewhat unclear and open to various interpretations. Hence, ethics curriculum in each medical school may vary according to teachers’ perceptions and how they interpret and contextualize the educational framework into their curriculum. Leite et al. suggested that contextualizing the national curriculum may correspond better to students and their local situations [3], while Marulcu and Akbiyik’s study showed that teachers often adopt the social reconstruction ideologies as their curriculum ideology to fulfil their perceived goals and personal beliefs of constructing a better society [4].

Previous studies have discussed the diverse views, scope and aim of ethics education, and the need of a consensus for a more homogenous curriculum in medical ethics [5–7]. However, since ethics is considered to be about values, and values are partly influenced by culture [8–10], we question to what extent the teachers’ perceptions concerning learning goals of medical ethics curricula are similar or different in Indonesia and the Netherlands, and if differences in goals are acceptable or considered problematic.

The first step of curriculum development should include problem identification and general needs assessment [11]. Previous studies have identified various perceptions as well as diverse needs and preferences of medical students towards ethics curricula [12], including the ethical problems they encountered during clerkship [13,14]. These studies are important to support data and information concerning students’ learning needs and to further develop an ethics curriculum that is most relevant and suitable to their respective conditions and contexts. Our previous study showed that there are similarities and differences between what Dutch and Indonesian students perceive as ethical problems and what they might need and expect from their ethics training [15]. However, we do not know from the teachers’ side, what they consider are the most important outcomes from ethics teaching and what they expect from students as future doctors through ethics education. This paper wishes to explore what Dutch and Indonesian teachers think are the important goals of ethics teaching, how they might be different or similar, and to describe and discuss the meaning of these differences and similarities in the development of medical ethics teaching globally.

This paper is part of a larger study on ethics education in the clerkship phase in Dutch and Indonesian medical schools. Historical ties between Indonesia and the Netherlands have paved the way for long-existing collaborations in education and research, including medicine and health. However, it has only been recently that the two countries have started to collaborate in the field of medical ethics education. The existing cultural, religious, social and educational structural differences between the two countries were most appealing for the authors to explore and compare the two groups. We believe that exploring and comparing the two groups of participants could bring more global understanding of ethics teaching and broaden teachers’ knowledge and perspectives, especially among teachers in countries with very different social and cultural backgrounds, such as Indonesia and the Netherlands.

## Methods

Our study used a phenomenological approach to explore the perceptions of ethics teachers in teaching medical ethics. We did not use any predetermined theory, nor did we intend to develop any new theory, but aimed rather to describe and discuss the experiences and perceptions of our subjects. We conducted in-depth interviews with purposive sampling. For the Indonesian setting, we collected names of teachers from our professional network of ethics teachers in medical schools, and from mailing lists of bioethics training courses and workshops. We then made a list of 25 teachers from 13 medical schools in Indonesia and selected 18 teachers with a diverse sample across teaching sites, demographic characteristics, and educational backgrounds. All 18 teachers who were invited agreed to participate, but one interview was eventually cancelled by the candidate due to competing tasks. After conducting 17 interviews, we found no new themes nor categories. However, we agreed to add three more teachers to ensure data saturation, and make sure that no new themes emerged. In total, we interviewed 20 teachers from 13 medical schools in Indonesia. In the Dutch setting, we invited 15 teachers from all 8 medical schools in the Netherlands. One teacher did not respond, and three others referred us to other colleagues. We then added two teachers to ensure data saturation, adding up to a total of 16 teachers from all 8 medical schools in the Netherlands. In total, we conducted in-depth interviews with 36 teachers (IDN = 20, NLD = 16) from both settings (Table 1). Interviews were conducted between January and September 2019.

**Table 1.** Teachers’ characteristics

Characteristics		Indonesia (20)	Netherlands (16)
Sex	Female	9	8
	Male	11	8
Home base university	Public	15	13
	Private	5	3
Teaching experience	<5 years	5	3
	5–10 years	8	4
	>10 years	7	9
Education background	Health (only)	16	-
	Humanities	2	9
	Health + Humanities	1	5
	Health + Humanities + Law	1	2

*Health: medicine, nursing, medico-legal

**Humanities: ethics, humanistic, philosophy, theology

We contacted participants through e-mails (Dutch setting) and text messages (Indonesian setting) and sent consent forms through e-mail before the interviews. Almost all participants were familiar with one of the research team members, which was valuable in gaining trust and building rapport. Interviews were mostly conducted at participant’s respective workplaces, two outside of their workplaces, six through telephone, and ranged from 40 to 120 minutes. We obtained permission to have the interview audio-recorded and to take field notes during the interview. We explained that all data were kept anonymous and unidentifiable to ensure the teachers’ privacy and confidentiality. In-depth interviews in Indonesia were conducted by AM, RBW, and DL in *Bahasa Indonesia*, while interviews in The Netherlands were conducted by AM and RBW in English. We used a semi-structured interview guide which was pilot tested to the first two participants in each setting. We asked participants to share their experiences in ethics teaching, in particular during the clerkship phase, and what were the expected goals or what they considered most important for students learning ethics. Specific questions included how satisfied they were with their teaching experiences, in achieving the expected learning goals, the barriers and facilitators, and the challenges they face in teaching ethics to medical students. Further questions were then formulated according to participants’ narratives and responses. The follow-up questions were meant to clarify the responses from participants and enhance the interviewer’s understanding.

Coding and thematic analysis of the Indonesian findings were conducted by AM and DL, while coding and analysis of the Dutch findings were done by AM and MH. Codes, sub-categories, and categories related to expected learning goals were grouped manually using Excel sheets and tables. Categories were derived from the data and were checked against each other and with the original data set. Potential categories and sub-categories were discussed together with MF, AU, and DW until consensus was reached. AM, DL, and MF are medical ethics teachers in Indonesia, while MH and DW are medical ethics teachers in the Netherlands. AU is a professor of research methodology and qualitative methods in Indonesia, and not involved in ethics teaching. Interpretations of transcripts, including the English translations, were sent to participants through e-mail to ensure their own meanings and perspectives are correctly represented. Two participants did not respond, and two others gave clarification and minor corrections with suggestions on the English translation.

## Results

### Teachers’ characteristics

We interviewed a total of 36 ethics teachers with various educational backgrounds and length of teaching experiences (Table 1). Most teachers were based in public universities. The private universities were originally affiliated to religious foundations, i.e., Christian, Catholic or Islamic foundations. We found a difference in teachers’ educational backgrounds between the two countries. Almost all Indonesian teachers were medical doctors without any formal training in the field of humanities, and seven teachers who were doctors had a subspecialty training in medico-legal, which is a subspecialty in medical forensics. Only two teachers in the Indonesian setting were not doctors and had formal training in ethics, philosophy, and theology. On the contrary, all Dutch teachers had a formal education in the field of humanities, while less than half of them had a background in medicine and nursing.

### Similar goals

We categorized teachers’ goals from each setting and found three similar goals: being professional, dealing with ethical problems, and being part of society. Being professional in both settings meant that students should have an ethical and professional attitude and behavior. Dealing with ethical problems includes being able to discuss ethical problems from different perspectives and give moral arguments and reasoning, while being part of society entails that students are able to blend into society and their working environment. Interestingly, we found different nuances within those similar categories (Table 2).

**Table 2.** Similar goals in medical ethics perceived by Dutch and Indonesian teachers

Categories	Codes	
	Dutch	Indonesian
Being professional	Professional attitude and identity Personal characteristics Moral sensitivity Being critical Reflection Speak up Integrity	Professional behavior Ethical mindset and attitude Collaborate with others Good communication Being good role models Being humane
Dealing with ethical problems	Identify ethical problems Different perspectives Discuss ethical problems Learn to cope with problems Moral decision and reasoning Emotional risks	Identify ethical problems Different perspectives Discuss ethical problems Solve ethical problems Moral arguments Ethical principles
Being part of society	Understanding environment Understanding workplace and culture Seeing the relevance in practice Being part of a larger society	Maintain local wisdom Cultural values Social justice Social responsibility ‘Doctors for the nation’

#### Being professional

In the Dutch context, reflection was considered an important part of being a professional doctor. One Dutch teacher spoke about character education, explaining that attitude, personal characteristics, and personal virtues are very important in the long term. He mentioned that students should be critical of their profession and discussed the importance of each person being responsible (assuming responsibility) when something goes wrong and not keeping silent. Students should be able to find a way to talk about challenging ethical and moral situations in a constructive and positive way. This view was also emphasized by another teacher who spoke about integrity and about having a speak-up culture.

We have some kind of reflection … what does it mean to wear a white coat … we want them to think … to reflect on themselves, because they are socialized in a very particular way as medical students, they have to behave in a certain way, and they start to behave in a certain way on the day they wear a white coat, and we reflect with them on what happens. There’s a lot of socializing, discipline, and power involved, and it’s hidden and implicit. (T008)

I think teachers should tell them about ethics and integrity, and I believe that human beings really know quite well what integrity is. We don’t have to spell it out. We want to have integrity, we want to have a speak-up culture, we want to have a learning community … It’s much more interesting to prepare them on being a person, being a professional for a life-long learning in knowledge and attitude. (T016)

Slightly different, Indonesian teachers spoke about the importance of good role models with regard to being professional, not only as a learning method, but as a learning goal. They talked about the strong hidden curriculum, the presence of many bad role models and how ethics teaching should compensate for that deficit. Hence, teachers said that teaching ethics can be very challenging and almost ‘impossible’, as stated by one of the participants. Therefore, they expect students to become good role models for their juniors, to be humane, well-mannered and have good communication skills, because patients also take notice of these important values.

One of the driving forces for bioethics education is role modeling. It is impossible … well, not impossible, but it is difficult for us to expect students to behave (ethically) and to give good moral arguments, when what they hear and see is morally incorrect. Here we explain things that are normative (good), then they return to the hospital and see those (bad) things again. Our fear is that students will not listen and just think ‘these ethics lecturers are just talking, that’s not what we see in reality’. This gap is really my main concern, because it will affect the educational milieu. (G007)

The teacher, who was a medical doctor himself, described his concerns about the difficult learning environment and his rather pessimistic view about teaching ethics in an ‘unethical’ environment. This concern was also shared by many other ethics teachers in the Indonesian setting, who were mostly medical doctors. However, there were some respondents who thought that having both good and bad role models is an important part of the learning process in ethics education.

I don’t think it really hinders. Students say there are still many doctors who can become (good) roles models, especially senior ones, who are now elderly. But this doesn’t mean that young lecturers cannot become role models, because students are actually more comfortable (interacting) with residents. Students also feel they can accept that real life is not always as ideal as they think. Sometimes they come to understand why a doctor does something (bad) … ‘maybe because he was tired and so on’ … and why a doctor ends up shouting at a patient, for example. (G016)

Sharing the same view with other Indonesian teachers, the teacher thought that reflection is an important part of ethics education. Observing bad role models, in particular, can be a useful learning method to reflect on how students themselves think and feel about this dilemma, to encourage thinking about why someone would act or behave that way and come to better understand the circumstances, while also keeping in mind that the behavior is unprofessional and something they should not do.

#### Dealing with ethical problems

This similar goal was more profoundly stated and described more often in the Dutch context compared to the Indonesian. Dutch teachers discussed the importance of taking distance from the case and to be aware of emotional risks, including moral distress and burnout, when dealing with ethical problems. For Dutch teachers, teaching ethics is not about solving ethical problems. One teacher stated that medical students tend to want to solve ethical problems because they are accustomed to problem-solving. He believes that it is more important to learn ‘to live’ and cope with a problem, try to deal with it as best as possible, and to accept that there might not be answers nor any solutions to the problem.

The prime goal is to get students accustomed to ethical discussions, even if there is not an answer; try to make them bearable and acceptable and agreeable for everyone that is involved. (T005)

For Indonesian teachers, dealing with ethical problems was often referred to in the context of finding a solution as the end goal. This is perhaps the most important difference within this category. One teacher discussed about the importance of understanding the basic moral principles in order to solve ethical issues that emerge in modern medicine. He stated that the medical code of ethics will not help much, referring to the fact that in Indonesia, the code is often used as the main reference when dealing with ethical issues.

I think, basically, there should be principle-based ethics, or the basic moral principles, because it is related to teleology and in accordance with advances in science and technology; while the code of ethics is more into deontology, to show what is good … So, if we only refer to the code of ethics, it will be frustrating because if there are advances in technology and a (ethical) dilemma, for instance a brain death case, we can’t solve it only with deontology. (G014)

#### Being part of society

The third similar goal is ‘*being part of society’*. Dutch teachers expect students to be able to understand their environment and culture, blend into society, and see the relevance of their ethical knowledge in practice. One Dutch teacher explained that teaching ethics is about making students aware of the context, and to help decide what is best for the patient and everyone who is involved.

I think in medicine there is always the question that doctors always … a lot of doctors always wonder: ‘Am I doing the right thing?’ … So you should try to involve local people in ethics teaching because they know the environment, they know the culture in which ethics take place and good ethics is not like ‘universal’ good ethics; good ethics is because it is in the context in where the people work and the patients are treated. (T010)

Another Dutch teacher mentioned that students should be prepared for their future work as medical doctors who will be dealing with people from different backgrounds. He continued by sharing one of his concerns, as well as a challenge, for ethics education in the Netherlands, that medical students might have some difficulty in understanding people with another background.

It (ethics education) gives them also a context … and it’s also (important), I think, to make clear to students that their future work, whether it is biomedical sciences or medical, is part of a larger society that has norms and values to live together as good as possible; and you cannot detach their future job from that. (T013)

In the Indonesian setting, teachers not only expect students to be able to blend into society, but further to have a sense of social responsibility and contribute to society. One teacher spoke about a doctor who built a humanitarian ship for people in the remote islands who have limited access to healthcare, so she felt deeply touched when one of her students decided to work in a remote area in Sumatra prone to earthquakes. She hoped that it was one of the results of their bioethics teaching, where they shared the motto *‘Doctors for the Nation’* with students before each class.

First semester (we focus on): academic integrity, then ethics, humanity, law, human rights, and finally the awareness (to give something for our country) … So, when we enter the class, we say: ‘Who are we? (And students reply) Doctors for the Nation!’ (G004)

Similar to many other Indonesian teachers, one teacher shared her deep concerns about the current situations in the medical training and healthcare system in the country that are far from ideal, and which students will face later as future doctors.

Students are taught the ideal things, but when they enter the hospital/clinical phase … (sighing and shaking her head sadly) … But of course (there is still hope) … Otherwise, what’s the point of our fight/struggle? (T009)

For Indonesian teachers, teaching ethics is part of a struggle, to fight for social justice and call upon students to stand up for patients’ rights, as well as healthcare workers, in a still-developing healthcare system. They expect students to maintain local wisdom and cultural values, as opposed to simply following western textbooks. This sentiment seems to be part of the nationalist view expressed by many Indonesian teachers in our study who felt that the current medical ethics education is very much influenced by western values.

### Different goals

We found four other goals that differed between the two countries, with two from each setting Tables 3(a,b). Many Dutch teachers emphasized the importance of *understanding one-self* and *learning from others* as being some of their main goals, while Indonesian teachers discussed the importance of religion and keeping one’s *faith and piety*, as well as *obeying rules* and understanding the law, as important goals in ethics education.

**Table 3.** **a**. Non-similar goals of medical ethics perceived by Dutch teachers. **b**. Non-similar goals of medical ethics perceived by Indonesian teachers

Categories	Codes
Understanding one-self	Self-reflection Personal identity Aware of own values Question own behavior Aware of different roles Discuss feelings and emotions
Learning from others	Share experiences Open for discussion Be in dialogue with others Be able to explain to others Understanding other people
Categories	Codes
Being faithful and pious	Religious values Religious behavior Blessing for others Noble professionalism Being faithful and pious
Following rules/standards	Medico-legal Obey the law Obeying rules Code of ethics Aware of regulations

#### Understanding one-self

This category was often emphasized by Dutch teachers and hardly mentioned by Indonesian teachers. Dutch teachers expected students to be able to self-reflect and to be aware of their own values. This includes discussing feelings and emotions and being able to question their own behavior. One teacher described how she triggers students during small group discussions to reflect on their own thoughts and values in order to understand themselves.

My goal is that they understand themselves, that they reflect on themselves also. I try to give back to what they say: ‘So this is what you’re saying … umm … what does this mean? What do you think about it?’ So, they examine their own thoughts. (T003)

Another teacher described that ethics is an experiential knowledge that involves both cognitive and affective aspects, namely feelings and attitudes. He stated that it is not easy to reason from a totally neutral perspective because one becomes more or less emotionally involved when they think about ethical problems, even if they did not experience the cases themselves. He suggested that getting ‘involved’ deeper into an ethical case might be useful to develop one’s moral reasoning.

Let me make it more practical. For me, it means that you always need to spend time on how it feels for you in the situation. How do you feel involved? What do you feel about people doing this or that … And that’s not the end point for the moral reflection, but as a starting point, because you can never do without it. (T006)

#### Learning from others

One teacher (T006) discussed further that there are two end goals of ethics teaching. The first is to have moral sensitivity, and second is to know what we should do and should not do. He explained that moral sensitivity can only be developed when we interact and are involved with people, and when we are open and willing to see that morals and values could be viewed differently by others. This view was also shared by other Dutch teachers who mentioned about learning from others as an important goal in ethics teaching.

It’s about group interaction … try to see if they can learn from each other. ‘Okay, I hear you say this, but you (another student) say something different. Okay, what’s the difference?’ It’s important that you can acknowledge why you found a connection on what you agree on, but also acknowledge the difference … ‘Okay, what is at stake for you in this situation, why do you find that so important?’ Or: ‘what is under pressure here that you become so angry with it?’ And make that (the values) explicit. (T006)

From the interviews, we can see that the two goals (*understanding oneself* and *learning from others*) are closely related and intertwined, and they were strongly emphasized by Dutch teachers. Although Indonesian teachers mentioned ‘different perspectives’, which might be closely related to ‘learning from others’, it was mainly used in referring to solving ethical problems as the main goal.

#### Being faithful and pious

This category was often emphasized by Indonesian teachers and hardly mentioned by the Dutch. One Indonesian teacher who teaches ethics in an Islamic University emphasized the role of religion and its position in ethics education. She explains that religion should be a basis for everyone to be able to have an ethical mindset. Furthermore, like religion, ethics should be a ‘blessing for all’, not only for an individual or a single group of people. Therefore, religious values are embedded within the ethics curriculum and often become an important reference during ethics discussion.

If we practice our religion correctly/properly, then our ethical reasoning should work; it’s not the other way around … ethics should be a blessing (Bahasa Indonesia: ‘rahmat’) for everyone, not only for oneself. (G006)

Another Indonesian teacher also stated that ethics teaching, in her position at a medical school in an Islamic University, is very much related to religion, not only to Islamic values but also other religions. One of the goals is to be able to perform *‘muhasabah’*, a form of religious reflection and self-evaluation in Islam, leading to acts of avoiding evil and doing good. During clerkship, medical students are required to perform *muhasabah* as a written task, to better prepare themselves in entering each clinical round. This tradition is similar and resembles the purpose of self-reflection in the Dutch context, except that it is done from a religious point of view.

We have a kind of ‘self-assessment’, more into religion, called ‘muhasabah’ (self-evaluation), hoping to see that they can do much more positive things towards the end of their clinical rounds. (G016)

Interestingly, this view on religious values as an important part of ethics education was not only shared by teachers who were based in religious universities, but also by teachers who worked in public universities in Indonesia. Some teachers stated that ethics teaching is often too much oriented to western values, which does not fit well with the cultural and religious values in Indonesia. They believe that ethical and religious values should not be separated and therefore they instilled religious values within their teaching.

#### Following rules/standards

Another different category shared by Indonesian teachers was about maintaining an obedient attitude towards following rules and standards, which was considered important as a safeguard to avoid unethical behavior and practices. One teacher who was a medico-legal specialist suggested that the ‘atmosphere’ and ‘mood’ (Indonesia: *‘suasana batin’*) of ethics in Indonesia is directed towards issues of malpractice, which is the main source and a common implication of most ethical problems in the country.

In reality, almost all ethical problems eventually become legal problems because that is the atmosphere here, … there are lots of malpractice. (G014)

Another teacher said that, proportionally, the need for legal competence (understanding the law and regulations) should be minimum for medical students. However, she stated that in Indonesia this might be a basic need and therefore a basic competence, because it is more concrete and easier to grasp than ethics. Ethics is considered more abstract and might entail different perceptions. She explained further that it is easier if people are given rules first, and once they get used to them, they would come to realize that it is necessary to be obedient to these rules and would obey them.

For the Indonesian context, medico-legal should be the basic (need) because it’s more concrete; there should be strict rules first before ethics. Once they realize that they need the rules, then regardless of being watched or not, people will not break the rules. (G006)

Indonesian teachers generally consider this crucial, especially in complying to the country’s health law and its medical code of ethics to prevent students, as future doctors, from falling into ethical and legal misconducts, which they considered are prevalent in the country.

## Discussion

### Similar goals: global or local?

Teachers from both countries shared three similar goals, two of which are mentioned in the respective frameworks: to be a professional doctor and to be able to deal with ethical problems [1,2]. Previous studies suggest that these are indeed the two main goals or views of ethics education shared in many countries [16,17]. However, there were slight differences in our study concerning what teachers consider as being professional. Dutch teachers emphasized professional integrity and how students, as future doctors, should be critical of their own profession. For Indonesian teachers, professionalism emphasized more on how to behave professionally and how to communicate with patients and families. Hence, Indonesian teachers are concerned about how students can perform or demonstrate their professional behavior, whereas Dutch teachers are more concerned about how students can develop their own ethical understanding and inner sense of professionalism.

On dealing with ethical problems, Indonesian teachers consider the four basic moral principles [18] as an important set of standards to solve ethical problems. This is also evident from the questions that appear in the national examination for medical students on the subject of medical ethics, where ethical questions often refer to these basic principles using multiple choices with one single best answer. Hence, it is common in Indonesia to find students in ethics classes asking what the right answer or best solution is to an ethical problem. Dutch teachers, on the contrary, are quite hesitant to use the word ‘solve’, because they suggest that ethical problems cannot be solved in the same way clinical problems are solved. Moreover, Dutch ethics teachers want students to learn and accept that not all problems can be solved and that students should learn to cope with the ethical problems they encounter.

The third similar goal is *being part of society*, which is rarely discussed in previous literature concerning medical ethics education. This is interesting, considering that the UNESCO’s bioethics core curriculum proposes 15 principles based on the Universal Declaration on Bioethics and Human Rights (UDBHR) [19], in which the last six essentially underscore the importance of being part of society, including solidarity and social responsibility (UDBHR articles 12–17). A recent study conducted by Torda and Mangos [20] in Australian and New Zealand medical schools also showed that besides the two main goals, namely ethical knowledge and reasoning, and attitudinal or behavioral development, other specific goals were mentioned, including notions of social justice. In future discussions in medical schools, it may be possible to consider not only cases concerning the patient-doctor relationship, but also concerning the doctor’s responsibility to the society as a whole. This possibility can serve as a starting point to include social justice as part of the goals of medical ethics education in a globalized yet struggling world with social disparities and inequalities in many parts of the world.

### Different goals: acceptable or problematic?

The importance of reflection in ethics education has been discussed in previous literature [21,22] and appears as one of the main goals of ethics education in the Netherlands and in other western countries [23,24]. Dutch teachers in our study often mentioned *reflection*, as part of *understanding one-self* and *understanding others*, as an important goal in medical ethics. In the Indonesian context, reflection was very rarely mentioned, and it was referred to in the context of contemplation or self-evaluation from a religious point of view. Hence, ethical reflection seems to be one of the main differences in the learning goals between the two countries, where it is common in the Dutch setting and less common in the Indonesian contexts.

Previous literature suggested that in many Asian countries, culture and religion remain important aspects in medical ethics education [25,26]. Although culture and religion (beliefs) were often discussed during ethics classes in the Netherlands, they are not embedded within the medical curricula as such in Indonesia. We found that the perceived goals from the perspectives of Indonesian teachers in our study appear to be in line with the first area of competence of the medical training framework, namely *‘noble professionalism’*, which serves as the basic foundation for medical education in the country [2]. Competencies under this area include: (1) Belief in God, (2) Moral, ethical and discipline, (3) Awareness and obedience to the law, (4) Social and cultural insight, and (5) Behave professionally. Hence, these ethics competencies appear to be clearly defined in the Indonesian framework. Moreover, *being pious* and *obeying the law* are two goals that differ between the two countries in our study and are unique for Indonesia.

In the Dutch context, ethics competencies are deduced or translated from all of the CanMEDS competence domains, namely: medical expert, communicator, collaborator, leader, health advocate, scholar, and professional [1]. Unlike the Indonesian framework, the ethics competencies in the Dutch framework are spread throughout the seven domains and there is more flexibility and freedom to interpret the competencies. Although Dutch teachers mentioned health law as an important source of knowledge for medical students, it is not part of the goals of ethics teaching as it is in Indonesia, even though they are often related and intertwined when discussing ethical cases [27]. In the Netherlands, teachings of ethics and law are often organized separately for practical reasons, unlike the UK, where teaching and learning of medical ethics, law and professionalism are integrated throughout the medical curricula [28].

The question arises if such differences in learning goals also occur in other countries and regions and what this means for the global audience regarding medical ethics teaching. Unlike previous studies conducted in the USA and European countries, where many similarities were found [29,30], a study by Miyasaka suggested that medical ethics teachings in the Asian region are more diverse, not only regarding the organization of the teaching programs, but also regarding its content. The differences were reflected by the historical and sociocultural contexts of the medical schools in the respective countries [31]. Other studies suggested that cultural differences are a substantial factor and have a strong impact in perceptions of ethical attitudes and ethical decision-making [32,33]. Considering the strong influence and relationship of culture and ethics [34,35], it would be logical and sensible to understand that ethics is both global and local. Ethical values and principles may be universal, but many of them are perceived and practiced differently according to the local cultural context. Therefore, we argue that differences in learning goals in ethics education worldwide is simply fair and common. It is an existing situation that is ‘a given’ or a reality, which we need to deal with wisely and thoughtfully.

Students as well as doctors and health care workers nowadays can easily communicate, thus making lectures and discussions easily accessible to a wider range of audience across borders and regions. However, discussing ethics with peers and colleagues across regions carries a risk of a ‘clash of culture’ or ‘clash of values’. Introducing and discussing major differences in learning goals, such as the importance of reflection and emotion in the Dutch setting and the importance of religious values in the Indonesian, to a global audience can be challenging. The bigger challenge, however, might be to learn and understand the subtle differences within the similar or ‘universal’ goals, such as in defining professionalism. For instance, being professional in the Dutch context might not be considered the same in the Indonesian setting, and vice versa. From our experiences in Indonesia, and as shown in the results of our study, learning ethical principles and values from a western perspective carries the risks of misperception, if not out-right rejection, from some teachers. However, this backlash or reaction should not hinder or become an obstacle for ethics teachers from different parts of the globe to come together and engage in discussion. We believe that being connected means creating more learning opportunities, to get to know and learn from each other, and not necessarily having to adopt or incorporate those differences into the local context.

## Strengths and limitations

The sample size for the Indonesian setting is relatively small compared to the Netherlands, which only has less than one tenth the number of medical schools in Indonesia (8 compared to 86 schools in 2018). Indonesian participants in our study mainly came from Java and only a few participants were from Sumatra, Kalimantan, and Sulawesi. Hence, we could have recruited a more diverse respondent population, although one third of all medical schools in Indonesia are located on the island of Java. Some translations from *Bahasa Indonesia* to English might have slightly different meanings and perceived differently by non-Indonesian readers, although forward and backward translations were carefully done. To our knowledge, our study is the first to explore this topic in the respective countries and to make a comparison between the two.

## Conclusions

Our study suggests that despite the similar goals in medical ethics shared globally, there might be differences in how teachers in different cultural contexts perceive the goals with their local values and how they translate the goals into the learning process. We believe that differences in learning goals are fair and natural, and therefore should not become a barrier for teachers among different countries and regions to communicate and collaborate for the development of medical ethics education. Understanding differences in learning goals, as well as differences in perceiving ethical values, could be an important goal for ethics teachers worldwide to broaden their knowledge and perspectives.
